# Identification of Laser Parameters Acting on an Axisymmetric Domain Using an Artificial Immune System

**DOI:** 10.3390/ma18122895

**Published:** 2025-06-18

**Authors:** Arkadiusz Poteralski, Jolanta Dziatkiewicz

**Affiliations:** Department of Computational Mechanics and Engineering, Silesian University of Technology, Konarskiego 18a, 44-100 Gliwice, Poland; jolanta.dziatkiewicz@polsl.pl

**Keywords:** microheat transfer, finite difference method, two-temperature model, artificial immune system, optimization

## Abstract

The paper presents the control of the ablated gap of required dimensions in an axisymmetric domain made of metal. For this purpose, two parameters of the laser interacting on this layer were identified, which means laser intensity and characteristic time of the laser pulse. A hyperbolic two-temperature model was applied. This is a model in which there are two coupled equations for electrons and phonons. The model was supplemented with appropriate boundary and initial conditions. The direct problem was solved using the finite difference method with a staggered grid. An artificial immune system was used for the identification process.

## 1. Introduction

The mechanical structure optimization is a class of challenging and time-consuming tasks [1]. To determine the value of the fitness function and/or its gradient, it is necessary to identify the global solution and to do multiple computations of challenging direct problems. Proposing optimization strategies based on immune system applications is one of the most crucial stages in creating bioinspired algorithms. Numerous methods, such as the boundary element method (BEM), the finite element method (FEM), and the finite difference method (FDM), can be used to handle boundary-initial value problems. In this paper, the finite difference method was used.

The goal of the optimization algorithm was to identify the values of laser parameters in order to obtain the expected depth and width of the gap in the material in the specific time that was evaporated as a result of the interaction of ultra-fast laser pulse.

The direct problem was calculated using the finite difference method with the staggered grid. The artificial immune system (AIS) was employed as an optimization algorithm in this paper.

The main motivation of the authors was to develop a mathematical model enabling the modeling of the ablation process and to create software that would allow for the control of specific ablation parameters of a given part of the material. Ablation is a very important technique used in many fields of science and industry, such as medicine, mechanical engineering, materials engineering, and space engineering. The example used in this article fits into material and mechanical engineering. Examples of applications in medicine include the following: the treatment of heart arrhythmias and the removal of tumors in various organs. In space engineering, ablation can be used in the optimal design of thermal shields. Therefore, creating a mathematical model of the ablation phenomenon and then algorithmizing it is a very important and new part of science. It is planned to further develop this topic by expanding the range of possible materials for which the ablation phenomenon will be applied (medical or space engineering examples). The second motivation during the work on this article was the possibility of using computational intelligence for automated control of parameters that determine the desired ablation. As part of the work on several optimization algorithms, AIS was selected, which, with appropriately selected parameters, guarantees that we find optimal solutions. Combining this type of bioinspired algorithm with an ablation calculation tool seems to be an innovative technique not used so far, which is confirmed by the literature review.

Natural phenomenon observations were used to design the AIS. A process found in biological immune systems serves as the foundation for the development of artificial immune systems [2]. In addition to positive and negative selection methods, they can use the clonal selection mechanism. The clonal selection mechanism is employed in this research.

The AIS algorithm was chosen due to some of its properties that were essential for the optimization process. The primary benefit of using the AIS is that it does not require any knowledge of the fitness function’s gradient. This algorithm’s other benefits compared with other heuristics methods include multidirectional search, the ability to work on potential acceptable solutions, high resistance to becoming stuck in local extremes, the ability to find multiple extrema at once (including local ones that are close to the global solution), and the fact that information about the objective function is sufficient for the optimization process. The disadvantage of this algorithm is the speed of the optimization process (which may be longer than other algorithms like PSO or a EA).

The paper [1] presents a comparison of the artificial immune system (AIS), particle swarm algorithm (PSO), and evolutionary algorithm (EA) employed in this work. Optimization of topology, shape, and material properties is presented [1].

Knowing its advantages and disadvantages, a comparison of its efficiency and effectiveness for several selected tasks (mechanical systems) is presented using the AIS algorithm. In order to optimize a reinforced rectangular plate, AIS and PSO were compared in paper [3]. Optimization is taken into consideration for the dynamically loaded plate that is reinforced by a frame-like structure made up of straight beams. The stiffness of the plate was maximized by searching for the best stiffener locations. Both algorithms discovered the same solution and were successful.

In publication [4], optimization of thermomechanical structures was made using bioinspired methods (AIS, PSO, and EA). The optimization problem was formulated as the minimization of the maximal value of the equivalent stress and the minimization of the volume and the maximal value of the temperature. The second option was the maximization of the total dissipated heat flux with respect to specific dimensions of a thermomechanical structure. The final solutions obtained after optimization were identical, but in terms of efficiency, this time AIS was faster.

In the paper [5] an application of AIS, PSO, and EA to the optimization of the stacking sequence of plies in composites is presented. All algorithms found the same solutions after setting their optimization parameters appropriately.

It was determined to adopt AIS in this article after weighing the benefits and drawbacks of each approach and considering several optimizations carried out with AIS, PSO, and EA. The development of such algorithms and their use in identification and optimization problems have been extensively studied in recent years. Many research institutions around the world are now developing and implementing artificial immune systems [6]. For instance, they tackled the classification and optimization problem using bioinspired methodologies. Query expansion problem [7], learning neural network [2,8,9], and multi-objective optimization [10,11] are only a few of the many works that the authors have written. AIS is now widely utilized to tackle a wide range of problems, including highly specialized ones such as determining and controlling a fuel–ethanol fermentation process, identifying and diagnosing heart illness using ECG analysis [12], and selecting the finest wiener equalizers [13]. Among other fascinating applications, the AIS can also be used to build evolutionary trees [8]. The Bayesian Artificial Immune System (BAIS), which is employed in works [14,15], is a variant of the “classic” artificial immune system [16]. Transportation systems also take advantage of the AIS [17,18]. A hybrid version of this strategy is used in publication [19] to attain faster convergence to the global optimum.

According to the literature review, the suggested technique of determining the laser parameter values to determine the anticipated depth and width of the material gap is a novel approach that complements numerous works about material property design. Combining FDM with bioinspired optimization algorithms like AIS is a novel method that yields quick and high-quality results.

The literature study concludes that while bioinspired approaches are applied during optimization for a range of engineering challenges, they are not utilized for the materials covered in this paper. All of the programs used to determine the values of laser parameters, including the optimization technique and the software for FDM structure analysis, were developed by the authors of this research. Making software based on AIS widely available and simple to use with any application to address issues based on altering design parameters was one of the primary goals of the development process.

In order to address the problem used to establish the objective function, a procedure for the exchange of data (PED) between an optimization algorithm (AIS) and any software is created. Article [20] describes what the appropriate files should look like in order to use AIS to optimize a given problem. This process enables the optimization tool to be quickly and readily integrated with any engineering calculation package.

This work presents the identification of laser parameters in a thin gold layer during the ablation process. The axisymmetric hyperbolic two-temperature model is applied. This two-temperature model is described by two differential equations coupled with the electron–phonon coupling factor *G*. One of the equations concerns electron temperatures, and the other concerns lattice temperatures [21,22]. The hyperbolic two-temperature model was validated with an experiment conducted on a thin metal layer made of gold. Appropriate initial and boundary conditions have been imposed for this model. The finite difference method with a staggered grid was used to solve this direct problem [23]. Heat fluxes were calculated for odd nodes, while temperatures were calculated for even nodes. However, the problem described in this article has not yet been solved using AIS.

The structure of this document is as follows. The two-temperature model for the axisymmetric domain is explained in Section 2. This model gives precise results for heat transfer on the microscale. Particularly for the ablation process, the thermophysical parameters presented in Section 3 are temperature dependent and extremely nonlinear. The method of solving the direct problem is described in Section 4. In Section 5, the principle of AIS operation has been described and explained. Next, the optimization problem and numerical example are presented in Section 6. Finally, concluding remarks are given in Section 7.

## 2. Microscale Heat Transfer

**Figure 1:**
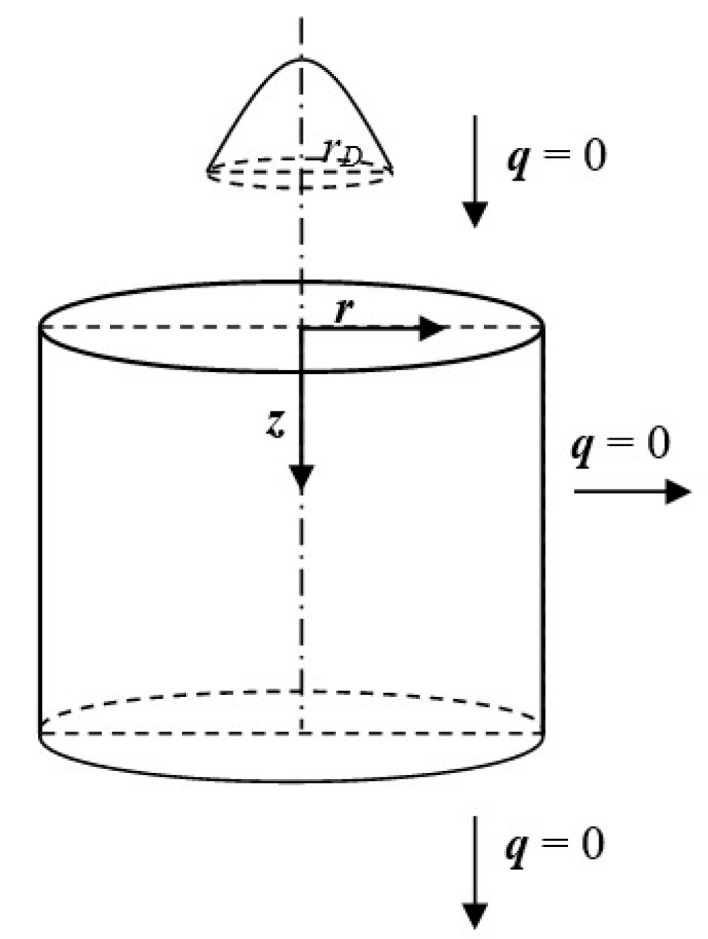
The axisymmetric domain.

The axisymmetric domain exposed to the ultra-short laser pulse is taken into account (Figure 1).

A two-temperature model that describes the isothermal solid–liquid and liquid–vapor phase changes as well as the temporal and spatial evolution of the lattice and electron temperatures in the irradiation microdomain has the form [21,24,25]

(1) $$C_{e}(T_{e})\frac{\partial T_{e}(r,z,t)}{t}=-\nabla \cdot q_{e}-G(T_{e},T_{l})\left(T_{e}(r,z,t)-T_{l}(r,z,t)\right)+Q(r,z,t)$$

(2) $$q_{e}(r,z,t+\tau_{e})=-\lambda_{e}(T_{e},T_{l})\nabla T_{e}(r,z,t)$$

(3) $$C_{l}\frac{\partial T_{l}(r,z,t)}{\partial t}=-\nabla \cdot q_{l}+G(T_{e},T_{l})\left(T_{e}(r,z,t)-T_{l}(r,z,t)\right),T_{l}\neq T_{m},T_{l}\neq T_{ev}$$

(4) $$\int_{t_{mb}}^{t_{me}}\left[-\nabla \cdot q_{l}+G(T_{e},T_{l})\left(T_{e}(r,z,t)-T_{l}(r,z,t)\right)\right]dt=Q_{m},T_{l}=T_{m}$$

(5) $$\int_{t_{bb}}^{t_{be}}\left[-\nabla \cdot q_{l}+G(T_{e},T_{l})\left(T_{e}(r,z,t)-T_{l}(r,z,t)\right)\right]dt=Q_{ev},T_{l}=T_{ev}$$

(6) $$q_{l}(r,z,t+\tau_{l})=-\lambda_{l}\nabla T_{l}(r,z,t)$$

where *T_e_* (*r*, *z*, *t*), *T_l_* (*r*, *z*, *t*) are the temperatures of the electrons and lattice, and **q***_e_* = **q***_e_* (*r*, *z*, *t*), **q***_l_* = **q***_l_* (*r*, *z*, *t*) are the heat fluxes of the electrons and lattice. *Q*(*r*, *z*, *t*) is the source function associated with the irradiation of the laser pulse, where the mentioned quantities are dependent on radial coordinate *r*, axial coordinate *z*, and time *t*. *C_e_*(*T_e_*), *C_l_* are the volumetric specific heats, *G*(*T_e_*, *T_l_*) is the electron–phonon coupling factor, λ*_e_* = λ*_e_*(*T_e_*, *T_l_*), λ*_l_* are the thermal conductivities of the electrons and lattice, respectively, τ*_e_* is the relaxation time of free electrons in metals it means the time needed to change energy state, τ*_l_* is the relaxation time in phonon collisions, ∇(∙) denotes the gradient, *Q_m_* is the volumetric latent heat of fusion, *T_m_* is the melting temperature, *t_mb_* and *t_me_* are the times corresponding to the beginning and the end of the solid–liquid phase transformation, *Q_ev_* is the volumetric latent heat of evaporation, *T_ev_* is the evaporation temperature, and *t_evb_* and *t_eve_* are the times corresponding to the beginning and the end of the liquid–vapor phase transformation.

Expanding the left-hand sides of Equations (2) and (6) into the Taylor series with an accuracy of two terms, one obtains

(7) $$q_{e}(r,z,t)+\tau_{e}\frac{\partial q_{e}\left(r,z,t\right)}{\partial t}=-\lambda_{e}(T_{e},T_{l})\frac{\partial T_{e}\left(r,z,t\right)}{\partial x}$$

and

(8) $$q_{l}(r,z,t)+\tau_{l}\frac{\partial q_{l}\left(r,z,t\right)}{\partial t}=-\lambda_{l}(T_{l})\frac{\partial T_{l}\left(r,z,t\right)}{\partial x}$$

The source function is related to the ultra-fast laser pulse, which has the form [26,27]

(9) $$Q=\sqrt{\frac{4\ln2}{\pi }}\left(1-R\right)\frac{I_{0}}{\delta t_{p}}\exp\left[-\frac{r^{2}}{r_{D}^{2}}-\frac{z}{\delta }-4\ln2\frac{{\left(t-2t_{p}\right)}^{2}}{t_{p}^{2}}\right]$$

where *R* is the reflectivity of the irradiated surface, *r_D_* is the laser beam radius, δ is the optical penetration depth, *I*_0_ is the laser intensity, and *t_p_* is the characteristic time of the ultra-short laser pulse.

When the lattice temperature *T_l_* reaches the value *T_ev_* at the point under consideration and condition (5) is met, the sub-domain associated with this point is eliminated, and the relevant boundary conditions are moved to the new external boundary. This is how the ablation effect is modeled.

The boundary conditions (no-flux conditions) are added to the mathematical model that was previously presented, which indicates

(10) $$\begin{array}{cc} \left(r,z\right)\in \Gamma : & \begin{array}{l} q_{be}\left(r,z,t\right)=-\lambda_{e}(T_{e},T_{l})\;n\cdot \nabla T_{e}(r,z,t)=0\\ q_{bl}\left(r,z,t\right)=-\lambda_{l}\;n\cdot \nabla T_{l}(r,z,t)=0 \end{array} \end{array}$$

where **n** is the outward unit normal vector.

The initial condition means that the initial temperature distribution is also known.

(11) $$\begin{array}{c} t=0:\;T_{e}\left(r,z,t\right)=T_{l}\left(r,z,t\right)=T_{0} \end{array}$$

where *T*_0_ is a constant value.

## 3. Thermophysical Parameters

**Figure 2:**
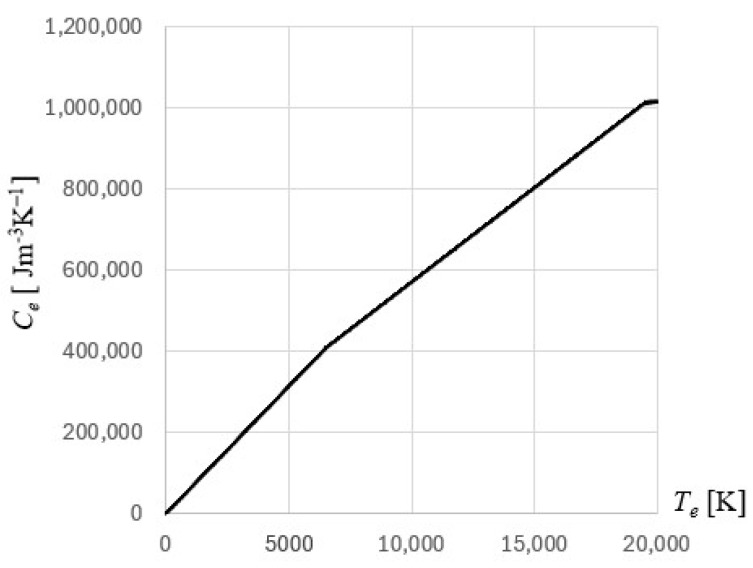
The course of volumetric specific heat of electrons for gold.

It is essential to accurately estimate the parameter values that exist in the model to guarantee the accuracy of the numerical computations. Volumetric specific heat of electrons depends on the temperature of the electrons, and the following formula is proposed [28,29]

(12) $$C_{e}(T_{e})=\left\{\begin{array}{c} \begin{array}{l} AT_{e},\;\;\;\;\;\;\;\;\;\;\;\;\;\;T_{e}<T_{F}/\pi^{2}\\ AT_{F}/\pi^{2}+\frac{Nk_{B}-AT_{F}/\pi^{2}}{2T_{F}/\pi^{2}}\left(T_{e}-T_{F}/\pi^{2}\right),\;T_{F}/\pi^{2}\leq T_{e}<3T_{F}/\pi^{2}\\ Nk_{B}+\frac{Nk_{B}/2}{T_{F}-3T_{F}/\pi^{2}}\left(T_{e}-3T_{F}/\pi^{2}\right),\;\;3T_{F}/\pi^{2}\leq T_{e}<T_{F}\\ 3Nk_{B}/2,\;\;\;\;\;\;\;\;\;\;\;\;\;T_{e}\geq T_{F} \end{array} \end{array}\right.$$

where *T_F_* = 64,200 K is the Fermi temperature, *N* = 5.9 · 10^28^ 1/m is the electron concentration in gold film, *k_B_* is the Boltzmann constant, and *A* for gold is given by the formula *A* = π^2^ *Nk_B_*/(2*T_F_*) = 62.7 J/(m^3^K). The course of this parameter in relation to the electron temperature for the gold film is shown in Figure 2.

**Figure 3:**
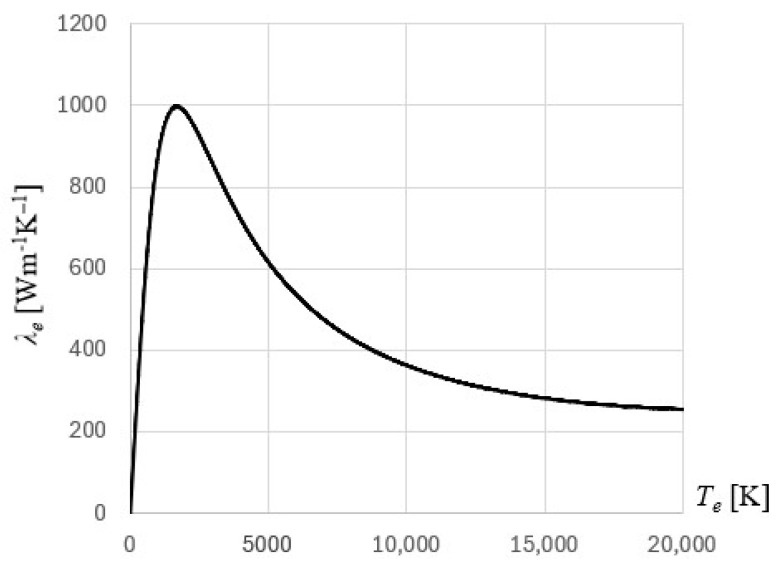
The distribution of electrons’ thermal conductivity for gold.

The electrons’ thermal conductivity is described by the formula [28,30]

(13) $$\lambda_{e}(T_{e},T_{l})=\chi \frac{{\left[{\left(T_{e}/T_{F}\right)}^{2}+0.16\right]}^{5/4}\left[{\left(T_{e}/T_{F}\right)}^{2}+0.44\right](T_{e}/T_{F})}{{\left[{\left(T_{e}/T_{F}\right)}^{2}+0.092\right]}^{1/2}\left[{\left(T_{e}/T_{F}\right)}^{2}+\eta (T_{l}/T_{F})\right]}$$

where for gold film one has χ = 353 W/(mK) and η = 0.16, [30]. The distribution of electrons’ thermal conductivity for the gold film is shown in Figure 3.

**Figure 4:**
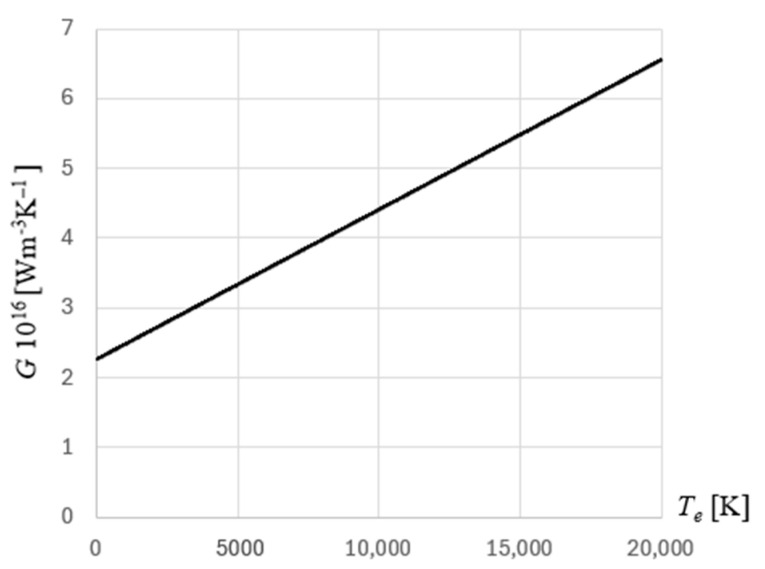
The distribution of the electron–phonon coupling factor for gold.

The electron–phonon coupling factor is given by

(14) $$G(T_{e},T_{l})=G_{rt}\left[\frac{A_{e}}{B_{l}}(T_{e}+T_{l})+1\right]$$

where for gold film *A_e_* = 1.2 · 10^7^ 1/(K^2^s), *B_l_* = 1.23 · 10^11^ 1/(Ks) and *G_rt_* = 2.2 · 10^16^ W/(m^3^K) [30]. The distribution of this parameter is shown in Figure 4.

The thermal conductivity and volumetric specific heat of the lattice are constant values [10].

## 4. Method of Solving the Direct Problem

The direct problem formulated is solved using the implicit scheme of the finite difference method [30,31,32]. In the cylindrical coordinate system, the Equations (1)–(3), and (6) take the form

(15) $$C_{e}(T_{e})\frac{\partial T_{e}(r,z,t)}{\partial t}=-\frac{1}{r}\frac{\partial \left[rq_{er}(r,z,t)\right]}{\partial r}-\frac{\partial q_{ez}(r,z,t)}{\partial z}-G(T_{e},T_{l})\left[T_{e}(r,z,t)-T_{l}(r,z,t)\right]+Q(r,z,t)$$

(16) $$C_{l}(T_{l})\frac{\partial T_{l}(r,z,t)}{\partial t}=-\frac{1}{r}\frac{\partial \left[rq_{lr}(r,z,t)\right]}{\partial r}-\frac{\partial q_{lz}(r,z,t)}{z}+G(T_{e},T_{l})\left[T_{e}(r,z,t)-T_{l}(r,z,t)\right],\;\;T_{l}\neq T_{m},T_{l}\neq T_{ev}$$

(17) $$\begin{array}{l} q_{er}+\tau_{e}\frac{\partial q_{er}}{\partial t}=-\lambda_{e}(T_{e},T_{l})\frac{\partial T_{e}}{\partial r}\\ q_{ez}+\tau_{e}\frac{\partial q_{ez}}{\partial t}=-\lambda_{e}(T_{e},T_{l})\frac{\partial T_{e}}{\partial z} \end{array}$$

(18) $$\begin{array}{l} q_{lr}+\tau_{l}\frac{\partial q_{lr}}{\partial t}=-\lambda_{l}(T_{l})\frac{\partial T_{l}}{\partial r}\\ q_{lz}+\tau_{l}\frac{\partial q_{lz}}{\partial t}=-\lambda_{l}(T_{l})\frac{\partial T_{l}}{\partial z} \end{array}$$

**Figure 5:**
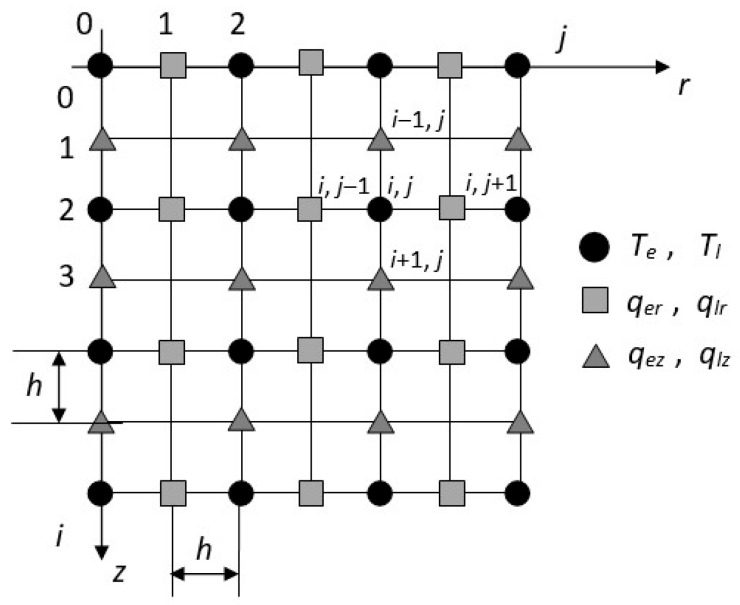
Staggered grid.

In this work, the finite difference method with the so-called staggered grid was used. The explicit scheme of the finite difference method requires that the stability condition be fulfilled, and for this purpose the mesh size has been appropriately selected. The nodes with any odd coordinates refer to heat fluxes, and nodes with even coordinates refer to temperatures, as shown in Figure 5.

The finite difference approximation of Equation (4) is used to represent the solid–liquid phase transition, as follows:

(19) $$\int_{t_{mb}}^{t_{me}}\left[-\frac{1}{r_{i,j}}\frac{r_{i,j+1}q_{lri,j+1}^{f}-r_{i,j-1}q_{lri,j-1}^{f}}{2h}-\frac{q_{lzi+1,j}^{f}-q_{lzi-1,j}^{f}}{2h}+G_{i,j}^{f}\left(T_{ei,j}^{f}-T_{li,j}^{f}\right)\right]dt=Q_{m}$$

For the node (*r_i_*_,_ *z_j_*), the value

(20) $$M_{i,j}^{f}=\frac{1}{Q_{m}}\left[-\frac{1}{r_{i,j}}\frac{r_{i,j+1}q_{lri,j+1}^{f}-r_{i,j-1}q_{lri,j-1}^{f}}{2h}-\frac{q_{lzi+1,j}^{f}-q_{lzi-1,j}^{f}}{2h}+G_{i,j}^{f}\left(T_{ei,j}^{f}-T_{li,j}^{f}\right)\right]\Delta t$$

is calculated, and it is assumed that *T*_*li*,*j*_^*f*^ = *T_m_*. For the transition *t^f^* → *t^f^*^+1^ the value *M*_*i*,*j*_^*f*^^+1^ is calculated. If *M*_*i*,*j*_^*f*^ + *M*_*i*,*j*_^*f*^^+1^ < 1 then *T*_*li*,*j*_^*f*^^+1^ = *T_m_* and the temperature field for transition *t^f^*^+1^ → *t^f^*^+2^ is determined. The calculations are continued until

(21) $$\frac{1}{Q_{m}}\sum_{k=f}^{K}M_{i,j}^{k}\geq 1$$

The phase change liquid–vapor is modeled in a similar way. Thus, if the lattice temperature *T*_*li*,*j*_^*f*^ is higher or equal to *T_ev_* (*T*_*li*,*j*_^*f*^ ≥*T_ev_*), then in the sub-domain domain *r* ∈ [*r_i_*_,*j*−1_, *r*_*i*,*j*__+1_], *z* ∈ [*z_i−_*_1,*j*_, *z*_*i*+__1,*j*_], the evaporation process starts. For this node, the value

(22) $$N_{i,j}^{f}=\frac{1}{Q_{ev}}\left[-\frac{1}{r_{i,j}}\frac{r_{i,j+1}q_{lri,j+1}^{f}-r_{i,j-1}q_{lri,j-1}^{f}}{2h}-\frac{q_{lzi+1,j}^{f}-q_{lzi-1,j}^{f}}{2h}+G_{i,j}^{f}\left(T_{ei,j}^{f}-T_{li,j}^{f}\right)\right]\Delta t$$

is calculated, and it is assumed that *T*_*li*,*j*_^*f*^ = *T_ev_*. For the transition *t ^f^* → *t ^f^*^+1^ the value *N*_*i*,*j*_^*f*^^+1^ is determined. If *N*_*i*,*j*_^*f*^ + *N*_*i*,*j*_^*f*^^+1^ < 1 then *T*_*li*,*j*_^*f*^^+1^ = *T_ev_* and the temperature field for transition *t*^*f*^^+1^ → *t ^f^*^+2^ is found. The calculations are continued until

(23) $$\frac{1}{Q_{ev}}\sum_{k=f}^{K}N_{i,j}^{k}\geq 1$$

Finally, the sub-domain under consideration is eliminated when the left-hand side of inequality (23) achieves the value 1, and the relevant boundary conditions are transferred to the new external boundary.

## 5. Optimization Method—Artificial Immune System (AIS)

**Figure 6:**
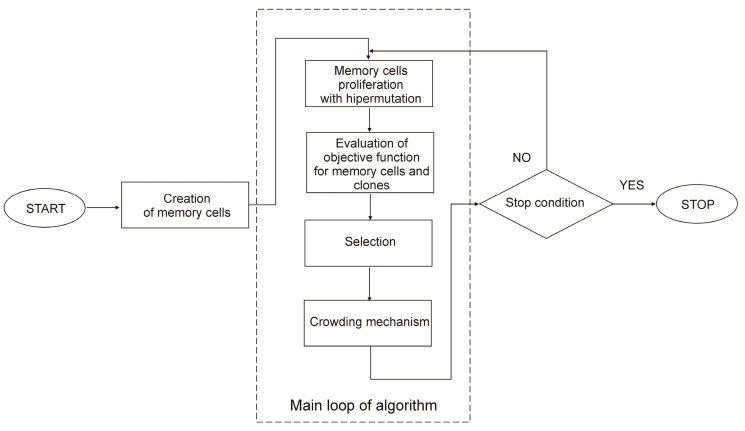
The algorithm of the artificial immune system.

An optimization approach like the Artificial Immune System (AIS) is used in this paper. AIS can be constructed using a variety of methods, including the mechanisms of positive and negative selection or the clonal selection mechanism [33]. This publication [34] explains the developed algorithm based on the clonal selection process. This method is shown in Figure 6. The memory cells are created at random in the initial stage. This process was carried out multiple times with different random values. This was a guarantee of the algorithm’s effectiveness. New memory cells are produced as a result of mutations and proliferation. The value of a memory cell’s goal function determines how many clones are produced from it. The objective function value of each memory cell is then ascertained.

Some of the new memory cells replace the memory cells from the previous iteration during the selection phase. The distance between memory cells and their clones, which is determined by applying the values of choice variables, is the basis for selection. The next step involves using the crowding mechanism. This process eliminates memory cells that are similar to each other. The process is carried out iteratively until the stop condition is met.

The stop condition can be represented by the maximum number of iterations, the expected value of the objective function, or the expected progress in the objective function over many iterations. This method alters the mutation operator and is based on the Wierzchoń method [35]. This approach substitutes the Gaussian mutation for the nonuniform mutation [35]. This type of mutation allows for the modification of previously obtained results in a particular manner. More information about this type of mutation can be found in [20]. Gaussian mutation aims to look for a certain memory cell’s surroundings. Several tests showed that this specific algorithmic mutation yields superior results to other types.

The objective function for the optimization problem is established. The task of the immune optimization is to minimize (or maximize) the objective function *J*:

(24) $$\underset{x}{\min J}(x)$$

regarding the decision variables, which are encapsulated in a vector:

(25) $$x_{t}^{j}=\left[g_{1}^{j},g_{2}^{j},\ldots ,g_{i}^{j},\ldots ,g_{n}^{j}\right]$$

where: $g_{i}^{j}$—*i*-th parameter of *j*-th vector, corresponding to the decision variable of the optimization problem, t—number of iterations.

Structure optimization occurs in a setting where constraints, including those on equivalent stresses, structural displacements, or structure volume, can be imposed. The decision variables, or vector (2) parameters, are then subject to restrictions:

(26) $${g_{i}^{j}}_{\;\min}\leq g_{i}^{j}\leq {g_{i}^{j}}_{\;\max}$$

where:

${g_{i}^{j}}_{\;\min}$—*i*-th minimal value of the parameter for the *j*-th memory cell,
${g_{i}^{j}}_{\;\max}$—*i*-th maximal value of the parameter for the *j*-th memory cell.

The initial stage of the immune process creates a group of memory cells. Memory cells, which are used to store decision factors, grow during the optimization process. The process of proliferation requires the creation of clones based on memory cells. The unique group for which the objective functions are computed is created by each memory cell and its clones. The creation of memory cells, the initial phase in the main loop of the immunological algorithm, is implemented in the next stage. Each memory cell has a set number of clones created for it. To find the ideal memory cell, this function creates the specified number of clones. For each additional memory cell, only half of the specified number of clones are created.

In this approach, the hypermutation mechanism is employed. The main duty of this operator is to ensure that fresh differentiated memory cells are formed. This approach modifies a single, randomly selected decision vector segment using a probabilistic method. Each decision vector position is altered with a specific probability using the following change $mut_{G}$:

(27) $$g_{i\_new}^{j}=g_{i}^{j}+\Delta g_{i}^{j},i=1,\ldots ,L_{pop\_kl}$$

where: $g_{i\_new}^{j}$—new value of decision vector parameter after using Gaussian mutation, $g_{i}^{j}$—*i*-th parameter of *j*-th decision vector corresponding to *i*-th decision variable of optimization problem, $\Delta g_{i}^{j}$—mutation with a random value of Gaussian distribution.

**Figure 7:**
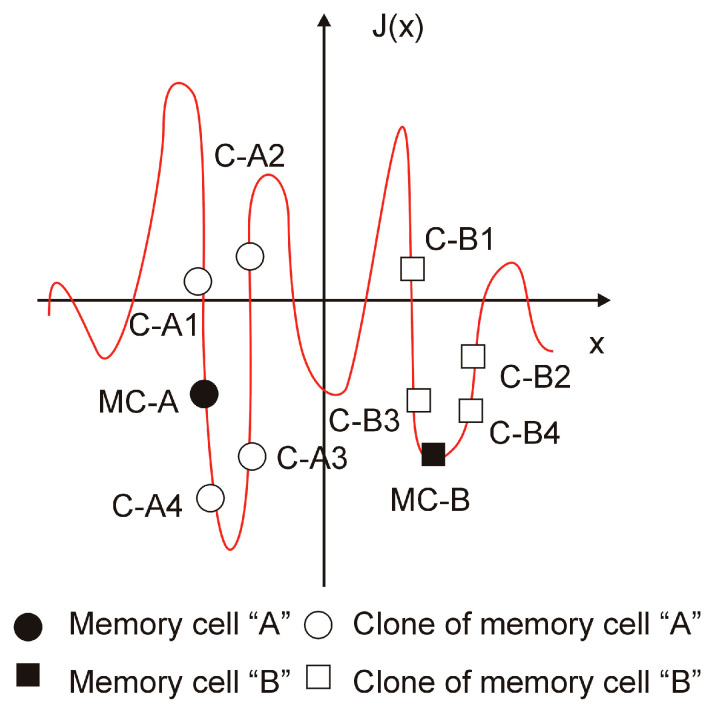
The idea of the selection mechanism.

In the next step of the AIS, the value of the objective function for every memory cell is determined. Selection aims to replace inefficient memory cells with more productive clones. During the selection process, some memory cells are replaced with better memory cells. The selection process uses decision factors to evaluate the geometrical distance between each memory cell and clones (Figure 7).

A description of the selection procedure based on the two memory cells “MC-A” and “MC-B” follows. Four clones are made for each of the memory cells “MC-A” and “MC-B,” ranging from C-A1 to C-A4 and C-B1 to C-B4, respectively. The clones of each memory cell are compared in terms of their objective function. In this comparison to the next iteration (better value of an objective function), clone C-A4 and memory cell MC-B are selected. This procedure must be repeated for each memory cell.

**Figure 8:**
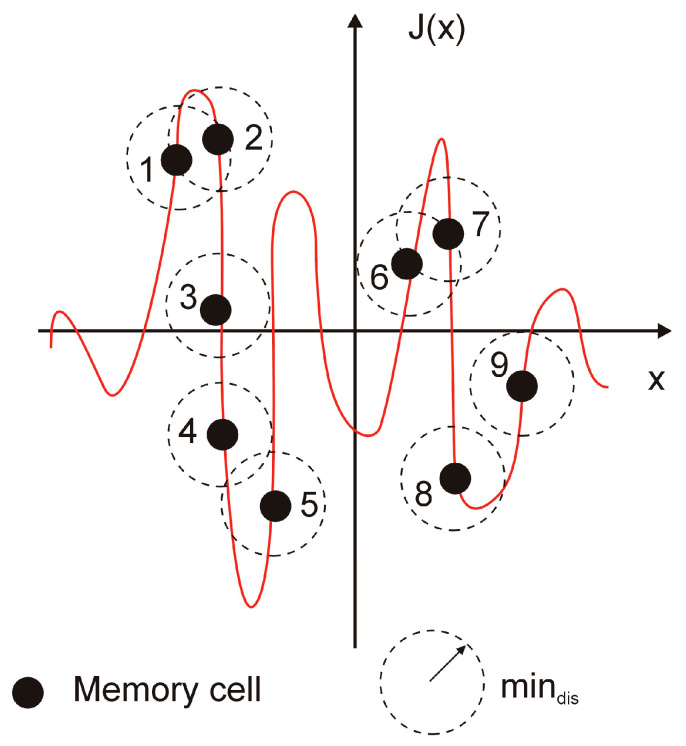
The idea of the crowding mechanism.

The final step of the immunological algorithm’s main loop makes use of the crowding mechanism. This mechanism is essential to the optimization process because it maintains the diversity of memory cells in the population. The crowding mechanism eliminates similar memory cells. Using the parameter min_*dis*_, the geometrical distance between two memory cells (Figure 8) is also used to calculate the similarity. During the crowding mechanism operation for memory cell “1,” only memory cell “2” is in the similarity area. This means that while the worst memory cell (“1”) is destroyed, the better memory cell (“2”) is kept in the population. Finally, a new memory cell is randomly generated. The other memory cells are not included in its similarity area. The population of memory cells still includes these memory cells.

The minimum distance between two points in the space of solutions $\min_{dis}$ is as follows:

(28) $$\min_{dis}=0.1\min_{dom}\left(\sqrt{\sum_{i=1}^{n}{\left({g_{i}^{j}}_{\;\max}-{g_{i}^{j}}_{\;\min}\right)}^{2}}\right)$$

where: ${g_{i}^{j}}_{\;\min}$—*i*-th minimal value of the memory cell parameter (decision variable), ${g_{i}^{j}}_{\;\max}$—*i*-th maximal value of the memory cell parameter, *n*—the number of memory cell parameters (decision variables), $\min_{dom\;}$—parameter deciding about the size of the search space of similarities of the memory cells.

The optimization process is repeated until the stop condition is fulfilled. The stop condition is described by the maximum number of iterations. The searched cell (the best memory cell) represents the unidentified global optimum.

The main advantage of the AIS is that it does not require any knowledge of the gradient of the fitness function and offers a high probability of finding the global optimum. More advantages of the algorithm include multidirectional search, working on possible acceptable solutions, high resistance to getting stuck in local extremes, finding multiple extrema simultaneously (including local ones near the global solution), and the fact that the optimization process only requires information about the objective function [35]. The disadvantage of this approach is the speed of the optimization process, which may take longer than other algorithms [36].

## 6. Results of Computations

This work aims to identify the values of laser parameters to obtain the expected depth and width of the ablated gap in the material in a specific time that was evaporated as a result of the interaction of an ultra-fast laser pulse. To evaluate the values of thermophysical parameters, an artificial immune system was used. These parameters occur in laser heating equations, which means *I*_0_ is the laser intensity and *t_p_* is the characteristic time of an ultra-short laser pulse. These two parameters allow us to control the heating process to achieve the expected depth of the gap at the specific moment.

For this purpose, a problem of optimization was formulated and solved using an artificial immune system. The value of the objective function determines the quality of the obtained solution. The objective function is based on the least squares method or the norm between the values of the numerical model and the expected value. The objective function has the following form:

(29) $$J(x)=\sqrt{\frac{\sum_{i=1}^{n}{\left(d_{i}(x)-d_{i_{ref}}\right)}^{2}}{n}}$$

where: **x**—is the design variables vector, *n*—is the number of measurements in sensor points, *d_iref_*—is the *i*-th expected value, *d_i_*(**x**)—is the *i*-th value obtained from the numerical model.

For the direct problem, the cylindrical domain (*R* = 100 · 10^−9^ m, *Z* = 100 · 10^−9^ m) of the initial temperature *T_p_* = 300 K (made of gold) is considered. Thermophysical parameters for gold are given in Table 1. The laser beam radius is equal to *r_D_* = *R*/8, and for gold, the reflection coefficient was R = 0.93 and the optical penetration depth δ = 15.3 nm [37]. To solve the direct problem, the finite difference method was used. Calculations are made for 50 × 50 = 2500 (*h* = 2 nm) ‘temperature nodes’, time step is equal to 0.002 ps.

There are two design variables identified: the laser intensity *I*_0_ and the characteristic time of ultra-short laser pulse *t_p_*. The limits on the values of design variables are given in Table 2.

The purpose of the identification is to obtain two modeled parameters. The identification is carried out based on the expected width, depth of the gap in the thin metal film of gold at a specific time. The series of calculations were performed for different values of the gap width and depth obtained at different times. The aim of the research is high precision of the ablation process. As a result of the presented calculations, the ablation process can be controlled with very high accuracy by changing the laser intensity and the duration of the laser pulse. There are three types of presented calculations. The first ones are where the identification is carried out on the basis that the expected width equals 14 nm in time 20 ps. The choice of such values of the gap width and depth is justified by the level of complexity of the calculations.

The parameters of the artificial immune system used for this example are given in Table 3. The 30 independent optimization processes were performed for the optimization parameters presented in Table 3.

**Figure 9:**
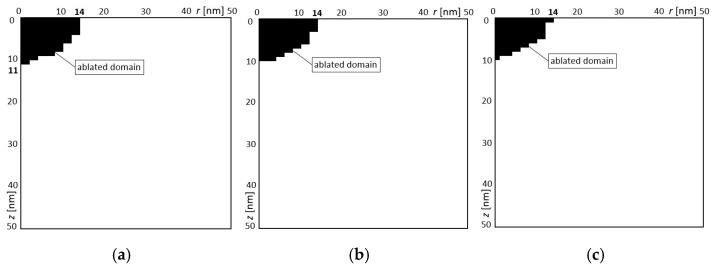
The obtained calculation results for the designed variables for the first example: (**a**) variant 1, (**b**) variant 2, (**c**) variant 3.

In the first example, three variants of design variables were obtained (Table 4). The solutions for variants 1, 2, and 3 were obtained 12, 8, and 10 times, respectively. These three obtained solutions are presented in Figure 9 and Table 4. Figure 9 shows half of the area in the *r* and *z* coordinate system. Since only the gap width was the optimization target, different variants were obtained for a larger or smaller gap depth. In summary, it is possible to obtain a constant gap width for the different depths of gap for different settings of laser intensity and ultra-short laser pulse. The main goal of the optimization was achieved, i.e., the specified gap width was obtained. The results of the numerical solution with the designed parameters are shown in Figure 9. The black domain means the ablated material. It can be seen that the expected width of the gap was achieved. There are only different depths of the gaps.

The second ones are where the identification is carried out based on the expected depth equal to 14 nm in time 20 ps.

The parameters of the artificial immune system used for the second example are given in Table 5. The 30 independent optimization processes were performed for the optimization parameters presented in Table 5.

**Figure 10:**
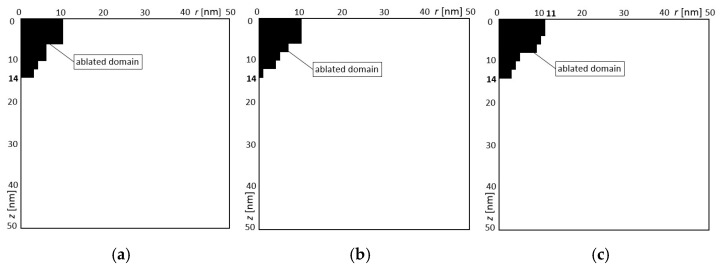
The obtained calculation results for the designed variables for the second example: (**a**) variant 1, (**b**) variant 2, (**c**) variant 3.

In the second example, three variants of design variables were also obtained (Table 6). The solutions for variants 1, 2, and 3 were obtained 6, 11, and 13 times, respectively. These three obtained solutions are presented in Figure 10 and Table 6. Since only the gap depth was the optimization target, different variants were obtained for a larger or smaller gap width. In summary, it is possible to obtain a constant gap depth for the different widths of gaps for different settings of laser intensity and ultra-short laser pulse. The main goal of the optimization was achieved, i.e., the specified gap depth was obtained. The results of the numerical solution with the designed parameters are shown in Figure 10. The black domain means the ablated material, as well. It can be seen that the expected depth of the gap was achieved. There are only different widths of the gaps.

The last examples are where the identification is carried out based on the expected depth and width equal to 14 nm in time 20 ps.

The parameters of the artificial immune system used for the last example are given in Table 7. The 30 independent optimization processes were performed for the optimization parameters presented in Table 7.

**Figure 11:**
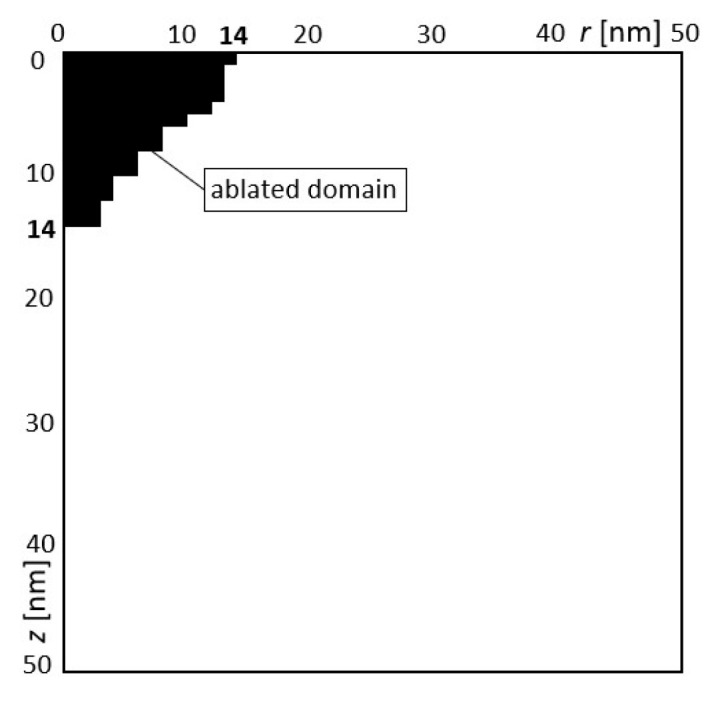
The obtained calculation results for the designed variables.

In the last example, the same variants of design variables were obtained for each optimized task (Table 8). The obtained solutions are presented in Figure 11 and Table 8. In a situation where the optimization goal is both the depth and width of the gap, which must be of an exact size, and no parameter could be arbitrary, only one solution was obtained. The main goal of the optimization was achieved, i.e., the specified area of the gap was obtained. The results of the numerical solution with the designed parameters are shown in Figure 11. The black domain means the ablated material, as well. It can be seen that the expected depth and width of the gap were achieved.

## 7. Conclusions

This paper presents a numerical model for identifying laser parameters in axisymmetric melting, evaporation, and ablation processes. These parameters, which were the laser intensity and the characteristic time of the laser pulse, were used in three tasks. The first task aimed to achieve the required width of the evaporated material in the required time. Using the artificial immune system optimization method, three variants of solutions were obtained. All sets of laser parameters provided the required gap width. However, differences in the gap depths can be seen in the results.

The second task was to achieve the required depth of the evaporated material in the required time. Using the AIS optimization method, three variants of solutions were obtained. All sets of laser parameters provided the required gap depth. Nonetheless, the results show variations in the gap width.

The last, third task aimed to achieve the required depth and width of the evaporated material in the required time and using the artificial immune system optimization method; one solution was obtained that provided the required gap dimensions.

Based on the obtained results, it can be concluded that the implementation of this approach utilizing AIS provides a high probability of finding the global optimal solutions. The AIS algorithm used in this paper has been successfully applied many times to optimize many physical tasks [1]. To summarize, the proposed method involving the use of AIS and FDM allows us to design new materials with new properties.

## Figures and Tables

**Table 1:** Thermophysical parameters for gold.

	Au
λ_0_ = λ*_l_* [W/(mK)]	315
*C_l_* [J/(m^3^K)]	2.5 · 10^6^
τ*_e_* [ps]	0.04
τ*_l_* [ps]	0.8
*T_F_* [K]	64,200
*T_m_* [K]	1336
*T_ev_* [K]	3127
*Q_m_* [MW/m^3^]	1229.989
*Q_ev_* [MW/m^3^]	32,771.400

**Table 2:** The design parameter constraints.

Parameter	Constraints
*I*_0_ · 10^6^ [J/m^2^]	1.5–3.0
*t_p_* [ps]	5–15

**Table 3:** The parameters of AIS for the first example.

Numbers of Decision Variables	The Number of Memory Cells	The Number of the Clones	Crowding Factor	Gaussian Mutation
2	5	5	0.5	0.5

**Table 4:** The design parameters for the first example.

Parameter	Optimal Value
variant 1—Figure 9a	
*I*_0_ · 10^6^ [J/m^2^]	1.345095
*t_p_* [ps]	9.410121
variant 2—Figure 9b	
*I*_0_ · 10^6^ [J/m^2^]	2.134337
*t_p_* [ps]	10.57472
variant 3—Figure 9c	
*I*_0_ · 10^6^ [J/m^2^]	2.000753
*t_p_* [ps]	10.50915

**Table 5:** The parameters of AIS for the second example.

Numbers of Decision Variables	The Number of Memory Cells	The Number of the Clones	Crowding Factor	Gaussian Mutation
2	5	5	0.5	0.5

**Table 6:** The design parameters for the second example.

Parameter	Optimal Value
variant 1—Figure 10a	
*I*_0_ · 10^6^ [J/m^2^]	2.110525
*t_p_* [ps]	10.79553
variant 2—Figure 10b	
*I*_0_ · 10^6^ [J/m^2^]	2.948634
*t_p_* [ps]	11.63030
variant 3—Figure 10c	
*I*_0_ · 10^6^ [J/m^2^]	1.174599
*t_p_* [ps]	9.350497

**Table 7:** The parameters of AIS for the last example.

Numbers of Decision Variables	The Number of Memory Cells	The Number of the Clones	Crowding Factor	Gaussian Mutation
2	10	10	0.5	0.5

**Table 8:** The design parameters for the third example.

Parameter	Optimal Value
*I*_0_ · 10^6^ [J/m^2^]	2.330836
*t_p_* [ps]	10.99929

## Data Availability

The original contributions presented in this study are included in the article. Further inquiries can be directed to the corresponding author.
